# High-reward, high-risk technologies? An ethical and legal account of AI development in healthcare

**DOI:** 10.1186/s12910-024-01158-1

**Published:** 2025-01-15

**Authors:** Maelenn Corfmat, Joé T. Martineau, Catherine Régis

**Affiliations:** 1 Faculty of Law, University of Montreal, Ch de la Tour, Montreal, QC H3T 1J7 Canada; 2 Faculty of Law, Economics and Management, University of Paris Cité, Av. Pierre Larousse, Malakoff, 92240 France; 3 Department of Management, HEC Montreal, 3000 chemin de la Cote-Sainte-Catherine, Montreal, QC H3T 2A7 Canada; 4 Canada-CIFAR Chair in Artificial Intelligence, Mila, St-Urbain, Montreal, QC H2S 3H1 Canada

**Keywords:** Artificial intelligence, Machine learning, Healthcare, Ethics, Law

## Abstract

**Background:**

Considering the disruptive potential of AI technology, its current and future impact in healthcare, as well as healthcare professionals’ lack of training in how to use it, the paper summarizes how to approach the challenges of AI from an ethical and legal perspective. It concludes with suggestions for improvements to help healthcare professionals better navigate the AI wave.

**Methods:**

We analyzed the literature that specifically discusses ethics and law related to the development and implementation of AI in healthcare as well as relevant normative documents that pertain to both ethical and legal issues. After such analysis, we created categories regrouping the most frequently cited and discussed ethical and legal issues. We then proposed a breakdown within such categories that emphasizes the different - yet often interconnecting - ways in which ethics and law are approached for each category of issues. Finally, we identified several key ideas for healthcare professionals and organizations to better integrate ethics and law into their practices.

**Results:**

We identified six categories of issues related to AI development and implementation in healthcare: (1) privacy; (2) individual autonomy; (3) bias; (4) responsibility and liability; (5) evaluation and oversight; and (6) work, professions and the job market. While each one raises different questions depending on perspective, we propose three main legal and ethical priorities: education and training of healthcare professionals, offering support and guidance throughout the use of AI systems, and integrating the necessary ethical and legal reflection at the heart of the AI tools themselves.

**Conclusions:**

By highlighting the main ethical and legal issues involved in the development and implementation of AI technologies in healthcare, we illustrate their profound effects on professionals as well as their relationship with patients and other organizations in the healthcare sector. We must be able to identify AI technologies in medical practices and distinguish them by their nature so we can better react and respond to them. Healthcare professionals need to work closely with ethicists and lawyers involved in the healthcare system, or the development of reliable and trusted AI will be jeopardized.

## Introduction

Recently, researchers, media, and practitioners have taken a keen interest in developments in artificial intelligence (AI). Indeed, since the launch of ChatGPT and GPT-4 by OpenAI at the end of 2022, citizens and professionals from all sectors, including healthcare, have been debating the contributions, impacts, and risks of such technologies. This paper outlines the main ethical and legal considerations associated with the development and deployment of AI within healthcare systems.

Medical doctors have used advanced technologies for many years. So why is AI different? *First*, it is far more disruptive. By allowing autonomous, opaque learning—and sometimes even decision-making—in a dynamic environment [1], AI leads to some unique technical, ethical, and legal consequences. For the first time since the birth of medicine, technology is not limited to assisting human gesture, organization, vision, hearing, or memory. AI promises to improve every area from biomedical research, training, and precision medicine to public health [2, 3], thus allowing for better care, more adapted treatments, and improved efficiency within organizations [4]. AI techniques including artificial neural networks, deep learning, and automatic language processing can now for example analyze a radiology image more quickly and precisely than a human [5], diagnose a pathology [6, 7], predict the occurrence of a hyperglycemia crisis and inject an appropriate dose of insulin [8], and analyze muscle signals to operate an intelligent prosthesis [9]. Yet, these improvements need to be balanced by the gap that now exists between the development (and marketing) of many AI systems and their concrete, real-life implementation by healthcare and medical service providers such as hospitals and medical doctors. This “AI chasm” [10] is notably explained by the disconnect that sometimes exists between the information technology (IT) side of system development and their adaptation to the specific needs and reality of healthcare institutions and patients, as well as by the ethical and legal issues discussed in this paper [10, 11]. Investment in the infrastructure that leads to AI solutions capable of “being implemented in the system where they will be deployed (feasibility), [and of] showing the value added compared to conventional interventions or programs (viability)” should also be targeted [12].

*Second*, health professionals generally seem to have rather poor knowledge of what AI is and what it allows [13]. While there is no unanimous definition of AI, the one proposed by the Organization for Economic Cooperation and Development (OECD) [14, 15] has gained international traction and is often referred to in various policy initiatives. Based on such definition, this paper includes all kinds of computational systems processing input data to generate outputs such as predictions, content, recommendations, or decisions that can influence its healthcare environment of implementation [16]. In healthcare, AI has great potential and it can be integrated to connected objects (e.g., smart blood pressure monitor [17]), robotic systems (e.g., surgical robot [18]), virtual assistants (e.g., patient management or appointment scheduling systems^1^), chatbots (e.g., customer service), contacts tracking during epidemic episodes [19], or medical decision support (e.g., radio image recognition for diagnosis^2^, choice of optimal treatment options^3^). The practice of medicine is based on medical doctors’ knowledge and experience, and AI’s dizzying calculation capacities mean that it can develop clinical associations and insights [20] on data derived from this knowledge (i.e., evidence from textbooks) and experience (i.e., lab results from patients) [21]. Thus, to the extent that the “AI chasm” can be reduced, healthcare professionals will increasingly see intelligent tools or machines being integrated into their daily practice [22]. This naturally provokes concerns such as the fear of being replaced and lack of confidence in the machine. In addition, healthcare professionals are poorly informed about the ethical and legal issues raised by the use of AI [23, 24].

Worries about the blind spots, complex implementation, impacts, and risks of AI have generated much political, academic, and public debates [15, 25]. Some have called for new ethical frameworks to guide the responsible development and deployment of AI, which has led to numerous declarations, ethics charters, and codes of ethics, proposed by organizations of every type [26], including international organizations [27], public and academic institutions [28], hybrid groups [28], and private companies such as Google [29], IBM [30], Microsoft [31], and Telia [32]. AI legislation has also been called for.

All these productions are sources of normativity [33]. In other words, they guide human behavior, providing parameters for what “should” and “shouldn’t” be done. However, the disciplines of ethics and law have distinct logics, conceptual frameworks and objectives and respond to different procedures of creation and implementation [34], making ethics and law two separate sources of normativity. First, law is composed of general, impersonal, external and binding rules, accompanied by potential formal sanctions (by courts or police for instance), while ethical norms do not exist in a coherent and organized set of norms – as in the case within a legal order - and while adherence to ethical principles is voluntary [35]. Second, legal rules derive from the state structure, in force at a given time, in a given legal space. The field of ethics, meanwhile, is derived from philosophy, and more recently social sciences, and relates to a reflexive process [36] that does not freeze ethical principles in time and space, but seek to define them in a more dynamic way. Third, legal rules seek to provide a framework for the coexistence of people in society, to protect its members and to guarantee political, economic and social interests at the same time, whereas ethical norms and discussions are more based on moral values [35]. In sum, legal rules could be defined as the minimal duty that every person must respect (whether one *can* do something), while ethics encourages reflection on choices and behaviors (whether one *should* do something). In healthcare, ethics first dealt with the manipulation of living organisms through “bioethics” before considering patient relationships through “clinical ethics” and management and governance through “organizational ethics” [37]. The latter two aspects are still difficult to grasp today, because they demand a global understanding of organizations that encompasses employees’ issues beyond the relationship of care.

Interestingly, despite the wealth of literature on AI, there is little to show healthcare professionals the main issues with an eye on the conceptual differences between ethics and law. This confusion is important to clarify, considering the different level of opportunities and limitations they bring forward in medical practice. Therefore, in this paper, we highlight how ethics and law approach the issues of AI in health from different perspectives. While law is mostly a local matter, our reflection does not target any one national jurisdiction. Nevertheless, the examples we use to better illustrate our analysis are focused on western countries and regions most active in the AI field (on the governance and technical sides) [38], i.e. the United States, Canada, Australia, the European Union and the United Kingdom. In ethical matters, the discussion encompasses a variety of ethical work on AI [39], but the monopolization of the ethical debate by a few countries from the Global North [38] should be underlined.

This paper presents an overview of the main issues pertaining to AI development and implementation in healthcare, with a focus on the ethical and legal dimensions of these issues. To summarize these, we analyzed the literature that specifically discusses the ethical and legal dimensions related to AI development and implementation in healthcare as well as relevant normative documents that pertain to both ethical and legal issues (i.e., AI ethics guides or charters developed by governments, international organizations and industries, as well as legal instruments). After such analysis, we created categories regrouping the most frequently cited and discussed ethical and legal issues. We then proposed a breakdown within such categories that emphasizes the different - yet often interconnecting - ways in which ethics and law are approached for each category of issues. Finally, we identified several key ideas for healthcare professionals and organizations to better integrate ethics and law into their practices.

The paper is divided into six sections, corresponding to the most important issues associated with AI in healthcare: (1) Privacy; (2) Individual autonomy; (3) Bias; (4) Responsibility and liability; (5) Evaluation and oversight; and (6) Work, Professions, and the Job Market. In conclusion, we advance a few proposals aimed at resolving some of the highlighted issues for healthcare professionals.

## Privacy

In machine learning or deep learning models, the computational algorithm solves problems by seeking connections, correlations, or patterns within the data on which it is “trained” [40]. Since the effectiveness of these models depends heavily on the quality and quantity of training data^4^, one of the most common techniques in AI technology development is to collect, structure, and use as much varied data as possible [41]. In the healthcare arena, this data can take many forms - such as measurements of a patient’s clinical vital parameters, biological analysis results, or genetic characteristics [42] -, and is created and collected from a wide variety of sources, from traditional healthcare system activities to self-tracking of consumers using digital technologies (“quantified self”) [43, 44]. Thus, this type of data is linked to an individual or a group who is directly or indirectly identifiable or targetable. However, health data is much broader than most people realize, and can also cover diet, exercise, and sleep - all collected by private companies outside the health system through connected devices such as smartphones and smart watches. Considering the intimacy and sensitivity of health data and the many actors potentially involved, AI highlights the question of individual privacy.

### The ethics of privacy

From an ethical point of view, issues of privacy are rooted in conflicting moral values or duties. The very concept of privacy has been defined in many ways in the ethics literature, with its origine intertwined with its legal protection [45], so it can hardly be summarized through a single definition. In the field of health, the search for what is right or wrong, appropriate or inappropriate, commendable or condemnable [46–48] is an ancient reflection that constitutes precisely the foundation of biomedical, clinical, and research ethics [37, 46]. In a context where people reveal details of illness, pain, life, and death [46], respect for their privacy as confidentiality of their information, and protection of their care spaces, both physical and virtual^5^, from interference or intrusion (e.g., constraint, coercion and uninvited observation) is crucial. Without this assurance of secrecy, patients would be less willing to share intimate information with their doctor, affecting their care or the usefulness of research [50, 51]. Safeguarding confidentiality of health information as well as personal health choices is also crucial in preventing discrimination, deprivation of insurance or employment [52], emotional stress, psychological consequences of revealing intimate information, and erosion of trust, among others [53]. Thus, preventing the damage caused by a violation of privacy is a major moral imperative in medical ethics^6^.

However, this principle of privacy is confronted with the duty to disclose information, either for the direct benefit of the patient (e.g., sharing of information for better care, their reimbursement, their own self-physical protection), for the benefit of others or society as a whole (e.g., disclosure of a communicable disease [55], protection of other victims [56], medical research [57], etc.), or for the commercial gains of AI specialized companies [58] that can all claim a valuable moral interest.

This tension between individual privacy and disclosure for potential useful uses is exacerbated by digital innovation, data analytics, and AI for several reasons. First, reliable AI development depends on access to health data, but this is restricted by the imperatives of confidentiality. Second, creating and using AI algorithms implies finding correlations across data sets that can allow the re-identification of individuals [2, 59], even if the data was initially anonymized [59]^7^, which could cause breaches of confidentiality. Third, the more the data is anonymized, the greater the risk that its utility is reduced. In addition, the portability and diversity of information collection systems (e.g. health, sport, or wellness applications; connected devices; data shared on social networks) make it much harder to guarantee the protection, security, and confidentiality of personal data [61] in comparison to data collected through the traditional health system (e.g., hospitals, clinics)^8^. For example, data that might initially be loosely related to someone’s health (e.g., daily calorie intake) can become more sensitive when correlated with other variables (e.g., a person’s weight), which is almost inevitable in the construction of an AI model^9^. However, taking this kind of data into account can help reveal more factors of a disease, and allows for a more predictive and personalized medicine^10^. These arguments all come as challenges to the principle of privacy.

Others take a very different view, departing from the principles of bioethics and privacy protection. For instance, engineers might argue that the astonishing recent advances in computing power, data collection, and the speed and ease of data exchange are realities that make privacy an outdated concept unsuited to our time^11^. In that sense, engineers may see privacy as a hindrance to the profitability of business models and innovation [53], thus limiting the benefits to health.

### Privacy and the law

From a legal perspective, privacy refers to the principles, rules, and obligations embedded in law that protect informational privacy and personal information. These rules are also challenged by the characteristics of AI techniques in the field of healthcare. Specifically, it becomes harder to respect principles and rights already enshrined in law, and the application of certain rules is more perilous - either because it ends up blocking the creation or use of a system, or because it does not allow the protection of privacy. While the following discussion is not exhaustive, it represents the bulk of legal discussions about informational privacy.

First, a law’s scope of application has a major impact on the protection that it will grant. While the common meaning of “personal data” may be clear^12^, its legal definition can vary between countries (and even within them). For example, it may refer narrowly to data managed and held in a particular file or by a particular entity (e.g., the U.S. HIPAA Privacy Rule, which covers certain entities within the traditional health system [64], or the Australian Privacy Act, which applies only to health service providers [65]). It may also extend its protection to information that allows both direct and indirect identification (e.g., first and last name, social security number, address, phone number, race, identification key, depending on countries), and re-identification capacities (e.g., overlaying two sets of data to create a deep learning database for the AI system). An example is the new California Consumer Privacy Act, which includes “reasonable” possibilities for re-identification^13^. Laws can define personal health data as data that is medical by nature (e.g., a medical test result), by purpose (e.g., used medically), or by cross-referencing (e.g., crossed with other data, as in AI analysis, to provide health information in combination)—as it appears to be the case with the French Data Protection Authority [66] based on the European General Data Protection Regulation (GDPR) definition [67].

Second, AI also challenges rules regarding the collection, use, and disclosure of personal data. For example, the requirement to determine the purposes for which data will be used in advance is a fundamental tenet of many privacy laws^14^. Similarly, the legal obligation of proportionality, minimization, or necessity requires that data be processed only to the extent necessary for the purpose at hand. However, many deep learning models require large amounts of data without knowing its purpose or even necessity in advance [68]. These principles will probably need to be revisited or relaxed if legislators wish to allow the widespread deployment of AI.

Third, meeting the conditions of access to qualitative and exhaustive health data held and produced by health systems is often a long, arduous, and discouraging journey for researchers^15^. Pooling and managing this data to offer easy but controlled access requires additional legal imperatives on technical security, in particular against cyberattacks.

Fourth, health and data protection laws do already consider AI through the way data is used and the consequences for the individual^16^. For example, fully automated decision-making and profiling systems are increasingly subject to special rules through legislative amendments in specific situations. For instance, there may be a specific right to be informed of the use of profiling techniques (as in the new Quebec’s Act modernizing provisions as regards the protection of personal information [70–72] or the new California Privacy Rights Act^17^); fully automated decisions are prohibited when they cause harm to the individual (as in the GDPR); and the right to have the decision reviewed by a human can be problematic, as the reasoning behind the decision is not always fully comprehensible.

## Individual autonomy

The second issue is closely related to some of the considerations outlined above. Autonomy is one of the four key principles identified by medical ethics. The Greek terms *autos* and *nomos* mean “self” and “law, rule,” so “autonomy” refers to a person creating their own rule of conduct and having the capacity to act without constraint and make their own decisions [73]. Many western jurisdictions incorporate the principle that free and informed consent must be obtained for any medical examination, treatment, or intervention, based on both the ethical principle of autonomy and the legal foundation of the inviolability and integrity of the person [74]. This principle of autonomy, as well as the moral value it embodies and the regulation that frames it, are confronted with several characteristics specific to AI.

### The ethics of autonomy

First, the “black box” phenomenon can impair the autonomy of the person whose data is processed for AI purposes. Indeed, some machine learning algorithms (e.g., the “random forest” classification algorithm) and, among them, deep learning algorithms (e.g., neural networks) have a high variability of inputs and a complex data-driven operation (non-linear system, where interactions do not follow a simple additive or proportional relationship), making it difficult for experts, let alone the general population, to understand how and why an algorithm arrived at a result (which we refer to as “intelligibility”) [75]. Whether it is about the process of model generation or the result obtained, the challenge is to provide a satisfactory explanation tailored to the user or person affected by the result, thus increasing the “interpretability” of the AI system [75].

In the medical context, increasing importance is placed on patients’ co-participation in their care [54] and their ability to refuse care or request additional medical advice. In some circumstances, the use of AI can erode the patient’s autonomy (even if the democratization of AI can also enhance people’s autonomy in other ways, including by increasing access to, and interpretation of, medical information). It may be difficult, if not impossible, for a patient to challenge a decision if the health professional cannot clearly explain how or why they proposed a certain treatment or procedure. Thus, the use of opaque, unintelligible AI systems might resurrect a certain medical paternalism, accentuating this loss of autonomy [76]. Refusing the use of the AI system may also be ethically questionable because of the characteristics of informed consent. “Valid informed consent requires clear and accurate recognition of the situation, absence of coercion (physical or psychological), and competence to make decisions (or representation, in the case of minors and incompetent adults)” [47].

Each of these three elements, however, differs depending on the individual’s level of AI literacy and other subjective characteristics (i.e., psychological, cognitive, or contextual), the interpretability of the algorithm used, and the amount and accuracy of information given to the patient. Currie and Hawks consider that “the public and patients are not always sufficiently informed to make autonomous decisions” [54]. Using nuclear medicine and molecular imaging as examples, they argue that people are probably underinformed and underqualified to determine what they want from AI, what they can expect from it, and thus whether they will allow AI to decide on their behalf [54]. Moreover, freedom to consent is called into question when access to a health service or the use of a connected tool is conditional on sharing personal data [77, 78]. However, maintaining trust in the use of AI in healthcare may push towards disclosing the use of AI for purposes other than treatment. In this regard, Amann et al. believe that “appropriate ethical and explicability standards are therefore important to safeguard the autonomy-preserving function of informed consent” [60].

Secondly, some controversial business practices reduce people’s moral agency, i.e. their ability to make moral choices, to exercise a form of evaluative control over them, and be held accountable for these choices [79], which impacts people’s autonomy. Tools ostensibly sold for healthcare or fitness (e.g., smart watches) become monitoring and information-gathering tools for the firms that collect these data [80]. These personalization technologies allow a “better understanding of consumer behavior by linking it very precisely to a given segment based on observed and inferred characteristics” (our translation) [81]. For example, “dark pattern” practices trigger the brain system that corresponds to rapid, emotional, instinctive, and routine-driven choice, producing an emotional stimulus that tips the consumer towards a purchase [81]. Thus, personalized manipulations join personalized prices in the marketer’s toolbox [81]. On the one hand, the user’s range of choices is narrowed according to their past consumption or the customer segment that the algorithm assigns them to (e.g., filter bubbles, misinformation [77, 81]). On the other hand, the commercial entity manipulates consumer behavior to create an incentive to purchase or consume a particular product (e.g., dark nudges, emotional pitches, or “dark sludge”^18^) [81]. The probability of a consumer being manipulated depends on their tech literacy and ability to spot the manipulation. These impediments to autonomy speak to the primordial moral and ethical choices of what constitutes a dignified, free, or satisfying human life, and several authors have exhorted us to deeply reflect on them [83].

Third, healthcare professionals’ autonomy may also be impacted, either because they use, are assisted by, or could be replaced by AI systems, which may have an impact on the delivery of care. The key players involved in the healthcare relationship need to maintain the agency over their actions, and the dilution of responsibility deserves to be thought through [80]. Conversely, “imposing AI on a community by a profession or a part of it is perhaps not ideal in terms of social or ethical standards” [54].

### Autonomy and the law

On the legal front, obtaining individuals’ specific, free, and informed consent is considered one of the ultimate expressions of autonomy [84]. Informed consent is usually required before personal information is obtained or used, either as a principle prior to any exchange of information – as in Quebec (Canada), for example [71, 72] – or as a legal basis on which to rely, as in the European Union^19^ or United States^20^. This relates to both the creation of an AI model and the context of its use in healthcare activities. Emerging issues include whether informed consent to care includes consent to the use of AI systems, machines, or techniques within such care [85]. Each jurisdiction makes a different choice, and each one is open to question. In Quebec, for example, the right to be informed must specify the professional who performs the therapeutic intervention [86], but not necessarily whether they used AI to make the diagnosis.

Inspired by the ethical reflection defining the contours of valid consent, the law usually requires that the person giving consent is sufficiently informed to decide in an objective, accurate, and understandable manner. In healthcare contexts, it usually encompasses information about the diagnosis, the nature and purpose of the procedure or treatment, the risks involved, and possible therapeutic options [86]. In addition, when personal information is used to make a decision based exclusively on automated processing, there is now a tendency to require data subjects to be informed of the reasons, principal factors, and parameters that led to the decision^21^. These requirements raise questions when using complex machine learning algorithms: the main factors and parameters may be difficult to report in an understandable way and raise questions about legal compliance [60, 87]. Informed consent may therefore be impacted, calling compliance with this obligation into question.

Second, valid consent usually implies that consent is obtained without pressure, threat, coercion or promise. However, patients rarely read or check the requirements for obtaining electronic consent, especially when it comes to personal information [88, 89]. The legal discussion ultimately concerns the possibility of respecting these requirements as well as other possible legal bases (e.g., another mode of consent), perhaps based on the notion that the subject’s autonomy resides more in general trust and transparency around AI use than in a button they unthinkingly click about 20 times a day [90]. In these questionable cases, an underlying ethical reflection supports research into solution strategies and the practical implementation of new legal requirements.

Finally, respect for autonomy also lies in the capacity to exercise the rights granted in principle to individuals [77]. This question deserves to be asked, in view of the characteristics of data exchanges and computer access that condition the construction of an AI system. The operation of certain AI systems may hinder people from exercising their right to be forgotten, their right to know what data is being used and what for, their right to limit the use of their data, the right to opt out, or the right to human review^22^—at least in certain legal jurisdictions. How can one ensure the deletion of an item of data where initial consent had been given for its use, when one does not know whether and to what extent that item has influenced a decision taken by the system? How can the right to human review of an automated decision be guaranteed when the reasoning behind that decision is unintelligible? What is the scope of the right to dereferencing or deletion if AI can aggregate information from the results of multiple search engines?

## Bias

Algorithms’ reasoning is precisely induced and driven by the data they are trained on. As a result, it can reflect biases present in that data, which will in turn impact the algorithms’ results and potentially exacerbate inequalities and discrimination against marginalized communities and underrepresented groups.

### The ethical view of bias

Some authors have categorized the main types of bias induced by AI [92]. The first is replicating or exacerbating societal and historical biases already present in the learning data (demographic inequality), which can lead to self-fulfilling predictions [93] and disproportionately affect particular groups [94]. One study reports, for example, that “the use of medical cost as a proxy for patients’ overall health needs led to inappropriate racial bias in the allocation of healthcare resources, as black patients were erroneously considered to be lower risk than white patients because their incurred costs were lower for a given health risk state” [95]. Yet, such lower costs also illustrate the inequalities in accessing medical services for black populations. As healthcare delivery varies by ethnicity, gender, housing status and food stability [96–98], among other things, feeding an algorithm with such data can make one of these social determinants of health a salient factor in the outcome [68]. “Creating a tool from data that fundamentally lacks diversity could ultimately result in an AI solution that deepens healthcare inequities in clinical practice” [54].

The second type of bias relates to incomplete or unrepresentative data [95, 99], especially that which over- or under-represents a subgroup such as a minority, a vulnerable group, a subtype of disease, etc [54]. When the theoretical reference population is not representative of the target population for which the model provides a result, there is a risk of bias, error, and overfitting, which can exacerbate health inequalities. For example, “an algorithm designed to predict outcomes from genetic findings may be biased if there are no genetic studies in certain populations” [68]. The risks of developing certain diseases often depend on other factors such as sex or age, and failure to account for these characteristics in the baseline training data biases the prediction of disease risks in other types of populations.

The third type of bias can be induced by the designers of the system themselves through the decisions they make when setting certain variables, the data to be used, or the objective of the algorithm [92]. The ethical issues that arise concern, for example, the possibility of predicting and possibly adding parameters that were not initially present in the data to make it as accurate as possible to eliminate bias. For instance, should the HIV status [93] of a patient who has refused to provide this information be added to the training data? And before even reaching the bias-correction stage, it is crucial to ask whether a potentially biased system should be introduced when it is already known it can reproduce societal biases. Moreover, the tech world seems to focus on eliminating individual-level human bias and training developers^23^. As Joyce et al. are arguing, “sociological research demonstrates, though, that bias is not free-floating within individuals but is embedded in obdurate social institutions” so that “there are severe limitations to an approach that primarily locates the problem within individuals” [96].

### Bias and the law

When considering the issue of bias from a legal perspective, the primary areas affected are the right to equality and protection from discrimination. Biases can affect decisions taken with respect to individuals, who may be discriminated against based on non-representative data or because some of their characteristics are accentuated by the operation of an AI model.

Equal rights legislation is based on the idea that individuals cannot be treated differently because of any personal trait or characteristic such as race or ethnic origin, civil status (e.g., marital status, gender expression, age), sexual orientation, health or social condition, religious and political belief, etc.^24^ It generally prohibits differential treatment in similar situations such as service access, employment, or housing unless justified by particular circumstances or legal duties [100]. The law often focuses on the effects on the victim [100] rather than the fault or bad intent of the perpetrator.

Although definitions vary by jurisdiction, an AI system used to determine people’s entitlement to reimbursement based on their higher risk in terms of health costs (e.g., that would be indexed to age, race, sexual orientation, etc.) could constitute discrimination under most legal systems in which equality is protected [101]. Yet, the context and the nature of the AI system could make proof of discrimination extremely difficult: determining the criteria behind decisions is difficult enough for the designers of some complex machine learning systems, especially if they are autonomous and evolve over time. One can imagine how much more difficult it would be for the individual victim of discrimination, who must obtain access to the information used and to the parameters of the model, which at present frequently remain opaque.

## Responsibility and liability

AI algorithms can sometimes make mistakes in their predictions, forecasts, or decisions. Indeed, the very principle of such models’ construction and operation is fallible due to the *theory of complexity* [102]. The computer program that underlies an AI model comprises a certain number of operations that allow it to solve a given problem. The complexity of the problem can be evaluated according to the number of operations necessary to reach an exact answer [103]. For highly complex problems, no 21st -century machine can surpass the threshold for the number of operations required. The objective of AI programs that tackle such problems, therefore, is “to compute a reasonably correct solution to the problem, in a computation time that remains acceptable” [103]. AI researchers call this type of calculation a “heuristic.” The system cannot ensure absolute certainty in its results, but it can (or at least hope to) propose better predictions than a human in the same situation, especially the least experienced clinicians [104] and is therefore of major interest. Apart from this intrinsic complexity, many different types of error impact on the responsibility of the actors involved throughout the lifecycle of an AI system.

### The ethics of responsibility

A first type of error arises from initial coding errors made by the programmer of the model. Unavoidable human error means there is a chance of the model providing incorrect answers in use. So, what probability of error can be accepted in these systems, and proceed to implement them in our society?

The need to maintain the quality of training data throughout the model’s lifecycle may also incur other types of liability-related errors. For example, image recognition based on artificial neural networks is one of the most advanced fields in AI [104]. Modifying inputs, “in the form of tiny changes that are usually imperceptible to humans, can disrupt the best neural networks” [105]. Finlayson and co-authors explain that pixels may be maliciously added to medical scans in order to fool a DNN (deep neural network) into wrongly detecting cancer [106]. The quality and representativeness of data (see section on Bias) and the opacity of the system (see section on Autonomy) can also lead to errors with detrimental consequences.

The misuse of a system is also problematic. Users’ level of knowledge about AI might vary greatly, whether they are a health worker helping to triage patients in the emergency department, a medical doctor handling an AI-powered surgical robot, or a patient setting up a connected device to measure their physiological vitals at home. Moreover, users might decide to ignore the result that the system provides, either because they misread it or because they consider it too far removed from their own assertions. Intentional malice aside, how should the responsibilities of the actors involved be considered? Over the short term, “human in the loop” approaches are recommended so that medical doctors take responsibility for their decisions while using AI systems, including the way information is used and weighed [54]. But to what extent should medical doctors be held responsible if they are unaware of an initial error in the input data, if they do not know the computational process leading to the result, or if it is beyond their power to modify it? Should doctors be liable for harm even though the model itself contains an error hazard due to the sheer complexity of the problem? Should the final decisions in medical matters systematically depend on human judgment alone? It remains difficult to argue that systems that provide personalized health advice, diagnostic or clinical decision support rely solely on human interpretation [68]. However, should the victims of the various prejudices potentially caused by AI systems (patient refusing care, unfair access to AI, discrimination, prejudice linked to privacy or physical harm…) be able to claim compensation? Indeed, some consider that it is inappropriate for clinicians who use an autonomous AI to make a diagnosis they are not comfortable making themselves to accept full medical liability for harm caused by that AI [95].

For complex systems, some of which work with reinforcement learning, it is still hard to predict what experiences the system will encounter or how it will develop. Like Pesapane and co-authors, one can thus question whether it is the device or its designer who should be considered at fault [68]. Should the designer be considered negligent “for not having foreseen what we have called unpredictable? Or for allowing the possibility of development of the AI device that would lead it to this decision?” [68] Some believe that if an autonomous AI is used according to the instructions, ethical principles require its creators to take responsibility for the damage caused [95]. However, similar to what we mentioned with respect to the risk of losing a certain degree of human agency in some circumstances (see section on Autonomy), the *automation bias* - which refers to the tendency of clinicians (and people more broadly) to overly rely on assistive technologies like AI^25^, calls into question the extent to which human responsibility should be considered.

### Liability and the law

From a legal point of view, AI errors are generally linked to the harm suffered by the victim and its reparation. In criminal matters, however, the legal perspective also encompasses the attitude that one wishes to punish, or the protection of society and other individuals from a possible recurrence.

Regarding the role of health professionals, we can look at current medical liability regimes to consider how mechanisms for civil liability and compensation for damages can be applied to the use of AI systems in health, and whether they consider the particularities of the operation and context. For example, in many fault-based liability regimes, the victim must prove that (1) the practitioner was at fault, (2) there was a prejudice (i.e., damage or infringement of a person’s rights or interests), and (3) there was a direct and immediate causal link between fault and prejudice [107]. Medical doctors are usually under an obligation of means (for example, products and equipment used) and much more rarely under an obligation of results. So, to determine the fault, the judge asks whether a “reasonably diligent” [86] medical doctor conforming to the acquired data of science and placed in the same circumstances would have acted the same way.

Yet, since the use of AI in medicine is so novel, a common understanding of how a “reasonably diligent” practice would look might need to be determined. How far would one consider the level of literacy of the medical doctor in relation to the AI decision support system? A surgical robot carrying out routine sutures under the control of an AI system remains under a medical doctor’s supervision: to what extent does the safety obligation imply liability for damage occurring during the operation, which the doctor might have been able to prevent with better knowledge of the system? We argue that judges will minimally require a sufficient understanding of the AI tools that medical doctors and other healthcare professionals use, based on explanations provided by the system supplier. At present, however, this interpretation is mostly at judges’ own discretion, and to the best of our knowledge, there are no major case-law decisions that could guide us.

Moreover, the opacity of AI systems and the many actors involved in their development and implementation make it much harder to prove a causal link between the fault and the damage—and the burden of proof invariably falls on the victim’s shoulders. The patient must know that such a system was used as well as all the steps in the decision-making process if they are to prove that the medical doctor should, for example, have disregarded the recommendation, detected an initial bias, checked the inputs, etc [110].

## Evaluation and oversight

To minimize the risks of using AI in healthcare, we need to evaluate AI systems before they are marketed, implemented, and used, and monitor them through ongoing oversight, especially for those systems that represent a higher risk for patients.

### The ethics of evaluation and oversight

Beyond the medical ethics principle of non-maleficence, the protection and promotion of human well-being [111], safety, and public interest implies that “AI technologies should not harm people” [27]. This idea, presented as the second of the six principles established by the expert group mandated by the World Health Organization (WHO), implies that the control, measurement, and monitoring of the performance and quality of systems and their continuous improvement must be paramount in the deployment of AI technology [112]. All actors involved should probably be accountable for these aspects. On this theme, there are several elements that merit consideration.

First, pre-deployment evaluation of AI systems involves determining the criteria for their evaluation. Today, most systems are evaluated within the framework of existing authorizations, certifications, or licenses, such as those issued by national health authorities for medical devices. These authorities examine the product or technology according to criteria that mostly relate to effectiveness, quality, and safety. Scientific validity is paramount, but should it be the sole criterion for the use and deployment of AI systems? In particular, the likelihood and magnitude of adverse effects should be assessed. In addition, there should be an “ethical” assessment that considers both the individual and collective benefits and risks of the technology, as well as its compliance with certain previously validated ethical principles. For example, the UK’s Medicines & Healthcare products Regulatory Agency (MHRA), the Food and Drug Administration (FDA), and Health Canada have developed “good practice” that aims to promote “safe, effective and high-quality medical devices using artificial intelligence and machine learning.” This document currently seems to incorporate^26^ a more global consideration by also integrating ethical concerns over the deployment of AI systems [113].

Second, AI technologies must be monitored and evaluated throughout their use, especially “reinforcement” learning models that take advantage of the data that is continuously generated and provided to carry on training and learning [114]. This is precisely what the WHO advocates, in the name of a final ethical principle that its committee of experts has termed “responsiveness.” Designers, users, and developers should be able to “continuously, systematically, and transparently assess” each AI technology to determine “whether it responds adequately, appropriately and according to communicated, legitimate expectations and requirements” [27] in the context in which AI is used. It is necessary to consider how these standards can be assured, taking into account the procedures and techniques available to do so [68].

The “human in the loop” approach is often seen as part of the responsible development of AI technologies. Applied to system evaluation, it could take the form of establishing several points of human supervision upstream and downstream of the design and use of the algorithm [115]. Establishing such a guarantee, which can also be described as a “human warranty” [27] or “human control” [116], would make it possible to ensure that only ethically responsible and medically effective machine learning algorithms [27] were implemented.

However, the question remains open as to how this approach can be applied to technologies that require no prior approval or regulatory authorization process, in particular because they do not qualify as medical “devices” or “instruments.” Such technologies, which often monitor fitness, women’s hormonal cycles, sleep, or overall well-being, can still have harmful consequences. The companies developing and selling such products often make public commitments through so-called ethical declarations and charters or self-developed ethical quality labels. End users, who are rarely qualified to evaluate whether developers’ actions are in line with these statements, risk falling victim to the phenomenon of “ethics washing” [117] denounced by AI researchers [118], ethicists, and philosophers^27^. The repurposing of the ethical debate to serve large-scale investment strategies merits intense reflection followed by action by public authorities.

### The legal view of evaluation and oversight

From a legal point of view, the issues also concern the regulation of marketing. First, as previously underlined, the definition of AI is neither unanimous nor stable, and this complicates the legal qualification of AI tools [68]. Indeed, tools qualified as medical devices are usually subject to strict rules concerning their manufacturing process, safety, efficacy and quality controls, evaluations, and more. In principle, they have a medical objective, and these constraints are therefore linked to the risks they pose to users’ health and safety. So far, however, the legal definition of medical devices rarely expressly includes all kinds of AI systems, even though some may share many characteristics of certain qualified devices or incur comparable risks. For example, in the United States, some types of medical software or clinical decision support systems are considered and regulated as medical devices [119], but the FDA’s traditional paradigm of medical device regulation was not designed for adaptive AI and machine learning technologies [120]. The inadequacy of this traditional vision and the lack of clarity on the regulatory pathway can have major consequences for the patient [93]. For this reason, the FDA has been adapting over recent years by specifically reviewing and authorizing many AI and machine learning devices [120, 121] and plans to update its proposed regulatory framework presented in the AI/ML-based SaMD discussion paper [122], which is supported by the commitment of the FDA’s medical product centers and their collaborative efforts [123].

Second, the quality control and assessment of medical devices are not fully adapted to the growing and constantly evolving nature of AI systems, the safety and effectiveness of which may have to be controlled over time. Classical legal regimes seem to be failing to incorporate all of the realities of AI systems and are in need of revision [124]; “the law and its interpretation and implementation have to constantly adapt to the evolving state-of-the-art in technology” [124, 125]. While some authors are still questioning possible approaches to the regulation of innovation, some countries have already made their choice. On the one hand, over-regulation [68] could stifle innovation and impair the benefits that AI would bring [126]. Conversely, “over-autoregulation,” or leaving the market to regulates itself, would lead in the other direction, with companies deciding for themselves which norms to develop and follow, solving problems as they arise. Several countries have chosen to rely on risk-based approaches for specific regulatory-device schemes to encompass these challenges. For example, the European Parliament has recently voted for its new Regulation on Artificial Intelligence (better known as the “AI Act”), which defines four levels of risk, where the minimal risk requires a simple declaration of compliance and the maximum risk incurs a ban on use. The Canadian Artificial Intelligence and Data Act (AIDA) proposal also plans, if adopted, to regulate AI systems based on the intensity of their impact [127].

## Work, professions, and the job market

In the health sector, AI’s impacts on jobs and work concern medical practice, the delivery of care, and the functions overseen by non-medical staff.

### The ethics of transforming work

AI systems are destined to become part of medical practice and care delivery, if they have not done so already. For example, an AI system mobilizing image recognition can detect a tumor on a mammogram [128]. In orthopedic surgery, robots with on-board AI are capable of assisting and securing the surgical gesture and ensuring better postoperative results by integrating the anatomy specific to each patient [129]. However, if these kinds of tasks become more widespread, might AI endanger jobs or even replace health professionals, as is often feared in technological transitions [130]?

Healthcare systems, professionals, and administrators will all be impacted by the implantation of AI systems. The first impact consists in the transformation of tasks. The integration of AI is transforming professional tasks, creating new forms of work [131], and forcing a readjustment of jobs (e.g., changing roles and tasks, modifying professional identities, evolving of professional accountability). For the WHO, readjusting to workplace disruption appears to be a necessary consequence of the ethical principle of “sustainability” identified by the committee of experts on the deployment of AI. In particular, governments and companies should consider “potential job losses due to the use of automated systems for routine healthcare functions and administrative tasks” [27]. Image recognition, for example, makes radiology one of the most advanced specialties in AI system integration [132]. AI is now able to “automate part of conventional radiology” [133], reducing the diagnostic tasks usually assigned to the radiologist. The authors of the French strategy report believe that this profession could then “evolve towards increased specialization in interventional radiology for diagnostic purposes (punctures, biopsies, etc.) for complex cases or therapeutic purposes guided by medical imaging” [133]. The practice of electrocardiograms in cardiology [133] or that of dentists in their routine and laborious tasks [134] is already undergoing upheaval. The field of general medicine is also being impacted by applications available to the public, such as “medical assistant” chatbots that can analyze users’ symptoms and direct them to a specialist or pharmacist. In the case of minor ailments, such technologies de facto diminish the role of the general practitioner.

However, if the medical doctor profession is safe for now, the role of an ethical approach is precisely to set guidelines, which could correspond to the level of social acceptability among the population and professionals’ desire to hang on to certain roles or tasks. For example, the “human in the loop” approach, as well as the principles of non-maleficence and beneficence, imply thinking about when the medical doctor should intervene and how much latitude they have in the face of automation [14]. The profoundly human character of care is a major element in the debate concerning the restructuring of missions and professional pathways [131]. The opportunity to “re-humanize” healthcare is opened up by handing over certain tasks to AI systems and should be seized. For example, the Paro therapeutic robot, which responds to the sound of its name, spoken praise, and touch, is used in geriatric services in Japan and Europe and has received positive reviews from patients [135]. For nurses and care assistants, the integration of these robots would take some of the physical and psychological strain out of their activity. However, while implementing such a tool might help to address human resources shortages, it may only be desirable for certain populations and contexts. Moreover, it will, of course, come up against other existential, social, and cultural issues, e.g., the evolution of social ties and the acceptance of this kind of technology in different cultures.

The transformation of skills is another consequence of the introduction of AI technologies into medical practice. As with the influx of computers into the workplace in the 1990–2000s, healthcare workers must learn to work with, or alongside, AI systems [27]. In addition to knowing how to use the technologies, health professionals should be aware of the repercussions and issues “technical, legal, economic or ethical posed by the use of tools based on artificial intelligence” [131]. Here, a risk arises that is similar to those related to the computerization and digitization of medical records: the time spent on training and correct use should not be to the detriment of clinical time, which is rightly considered to be paramount.

However, whereas previous technological revolutions concerned lower-skilled workers, AI may herald the opposite [136]. AI can pose the risk of a future deskilling among healthcare professionals, especially by inducing dependence [137] or cognitive complacency [138]. The capacities offered by automating cognitive work that previously required high-skill workers might cause consequences such as altering clinical reasoning processes (e.g., reducing a clinician’s diagnostic accuracy). However, the use and application of AI itself require periodic refinements by experts, including medical doctors [137]. Radiologists’ professional networks allayed this fear by reducing the scope in which AI could enter while recognizing the potential benefits of automating more routine tasks and upskilling their roles overall [139]. In situations where the use of AI is preferred, there are several ways to mitigate the risks of deskilling. For example, Jarrahi and co-authors suggest that some “informating capacities” of AI systems (i.e., capacities beyond automation “that can be used to generate a more comprehensive perspective on organizational reality” [138]) could be used to generate “a more comprehensive perspective on organization, and equip workers with new sets of intellectual skills” [138].

The impact of AI should also be considered at the more global level of managing organizations and non-medical staff. Areas affected include patient triage in the emergency room and the management and distribution of human resources across different services. This is where organizational ethics comes in, with human resources management and social dialogue figuring as major concerns. Indeed, in the health sector, the layers of the social fabric are particularly thick, diverse, and interwoven: changes in a healthcare institution affect many, if not all, of its workers, with major repercussions in the lives of users and patients too. The care of individuals who interact with medical assistants or diagnostic applications is also shifting. Thus, such “evolutions, introduced in a too radical and drastic way, damage the social fabric of a society” [120]. Moreover, these transformations also blur the boundary between work and private life and alter the link between the company and its employees, both old and new [140].

In this respect, the deployment of AI technologies certainly implies the emergence of new professions, which must be properly understood. For example, new technical professions such as health data analysts, experts in knowledge translation, quality engineers in ehealth, and telemedicine coordinators, as well as professionals in social and human sciences such as ethicists of algorithms and robots are to be imagined [141, 142]. The construction of the organization’s ethical culture will depend in particular on its ability to identify areas of ethical risk, deploy its ethical values, and engage all its members in its mission [143].

### Transformation of work and the law

The transformation of qualifications questions the relationship between the medical professions and technology, as well as the legislative and regulatory obligations for training. Requiring the medical doctor to be able to explain or interpret the outputs of an AI model remains a legal issue as well as a significant challenge. The upheavals within certain professions may mean that their regulation must be adapted—as the regulatory framework for radiologists in France has already been modified, redefining the acts and activities that can be performed by medical electroradiology manipulators [144]. According to the National Federation of Radiologists, the move towards diagnostic interventional radiology mentioned above has already been integrated by the profession [133]. The High Council for the Future of Health Insurance speaks of the major task of “concentrating and developing the role of medical doctors in expertise and synthesis activities,” which will certainly require regulatory change.

From a legal point of view, this issue could also call into question the right to be treated or cared for by AI rather than a healthcare professional. The trend towards quantified self or personal analytics, where data analysis and measurement tools become more powerful every year, has given individuals greater knowledge on managing their health and sometimes implies a different understanding of themselves as patients within healthcare structures. Individuals’ awareness and use of AI services is also growing, despite fears. That considered, some demands for surgery might be best met by AI, particularly if it is safer, quicker, more efficient and more likely to succeed. And if cultural differences or social acceptability lag behind such demands [145], one might justifiably ask whether they should catch up. Could the right to choose one’s doctor be extended to include the right to access an “AI doctor”?

## Discussion

The issues raised by AI in healthcare take on different nuances depending on whether one speaks of them in terms of legal compliance, the ethical choices behind practices and decisions, or reflective processes integrated into professional practices. We propose three avenues of reflection to address such issues.

### Education and training

Many AI tools are intended to be used by healthcare professionals (e.g., risk prediction of future deterioration in patients [146], clinical decision support system [147]; diagnoses assistance tools from radiological images [148]). Therefore, these professionals must know about these tools, how they work, and their implications to ensure the quality, safety, and effectiveness of AI. In order to deploy AI while taking all this information into account, there is a need to increase the technical, legal, and ethical AI literacy of healthcare professionals [149]. We propose two main ways to achieve this.

First, basic AI training should be integrated into academic programs, where students are the future users of AI in healthcare [150]. A study in Canada revealed that more than half of healthcare students [151] either do not know what AI is or regard it as irrelevant to their field. In addition, few institutions cover the goals of AI in their educational programs [152, 153]. This is a missed opportunity to address misconceptions and fears related to AI and to raise awareness about ethical and legal issues associated with these systems. As Wiens et al. explain, successful training involves bringing together experts and stakeholders from various disciplines, including knowledge experts, policymakers, and users [93].

Second, continuing education on AI for health professionals should be integrated into health organizations and institutions [13, 110]. Apart from illuminating the use of digital tools and data and the internal workings of systems, this training would engage health professionals’ moral responsibility. Confronted with a situation involving moral values, ethical principles, or the application of legal rules, they would question themselves before mechanically applying their technical knowledge. They could then reflect on the ethical consequences of their actions, such as the use of a particular AI tool, depending on the context and the patient involved. Depending on the situation, professionals could refer to the ethical principles and standards defined within the organization, their deontological code or the ethics committee within their organization. These reflexes are not new among medical professionals, since medical ethics has been widely implemented in processes and practices. Moreover, the important regulation of the health sector already forces professionals to question the conformity of their practices to the law or to ethics. However, these mechanisms deserve to be adapted to the use of AI.

Such training is widely encouraged by institutions such as the American Medical Association [154], which supports research on how augmented intelligence should be addressed in medical education, or the Royal College of Physicians and Surgeons of Canada [155], which recommends incorporating this teaching into the curricula of residents [112]. We believe that the responsibility for integrating training is shared between professional bodies, healthcare institutions and academic institutions. Indeed, we believe that the issues we describe cannot be resolved unless accountability is shared in such a way.

### Support and guidance

The second, complementary theme concerns the accompaniment of health professionals in these new practices. This support would first involve the creation of an internal or external interdisciplinary committee to approve the implementation of new AI technology—a special authority for AI at the organizational or institutional level. Such a committee should include ethicists, AI engineers and developers, healthcare professionals, patients and health organizations administrators would make it possible to assess whether a given technology met predefined evaluation criteria, based on the ethical issues it triggers, before it could be used. It should also include a lawyer to resolve certain legal issues and stay alert to the evolution of the law, which is bound to change to integrate the particularities of this technology.

The committee would also ensure that the technology has been developed around the skills, expectations, interactions, or technical or organizational constraints of the user. This would force AI developers to work with potential future users (including both healthcare professionals and patients), from the design stage onwards. The criteria adopted by the committee would then be integrated throughout the creation of the technology, giving it the best chance of being approved and implemented in the safest, most efficient, most collaborative and, therefore, highest-quality manner possible. Unlike institutions that review systems for regulatory and legislative compliance and evolve in parallel, this ethical approval process would be the responsibility of the institution’s administrators, who would also be responsible for building bridges between developers and users.

### Tool adaptation

Another solution concerns the AI tool itself, whose interface must be designed to serve the user, taking account of the issues that arise for them and allowing them to play an active role in the system (for example, in terms of control, decision-making, choice of actions, etc.) [156]. Thus, the bridge between designers and users would make it possible to create an interface that is intuitive, ergonomic, transparent, accessible, and easy to use.

As we have seen, one of the objectives of training health professionals is to encourage reflective thinking, which is broader than mere concern for legal liability. Functionalities to trigger the desired “ethical reflex” should be integrated into the heart of the interface—for example, alerting the professional about the diversity or source of the data they are entering, or even about the result that the machine has returned. One could even envisage that these alerts be personalized: indeed, some systems know how to personalize alerts based on the information they have about the situation. Instead of alerting users about the contraindication of a drug prescription or how to complete an exploration [157], the interface could provide alerts on certain ethical considerations. For example, medical doctors entering symptoms into a diagnostic support system could be alerted when specific data points (as input) were atypical and could prove particularly sensitive in the operation of this algorithm. Keeping the approach focused on the user experience, these functionalities should be light enough to preserve the human-machine interaction and the ergonomics of the interface (meaning that tasks can be performed within a reasonable time).

Finally, feedback loops should be established, coupled with the obligation for the professional to report any problems that occur when using AI. This functionality would prevent the professional from implicitly trusting the tool and force them to remain alert and critical regarding its recommendations, predictions, previsions, or other results.

### Limitations

We have tried in this paper to present an encompassing view of the ethical and legal issues surrounding the development and implementation of AI in healthcare. However, we recognize that our research has limitations. First, the six issues presented are not exhaustive since they include those most cited in the targeted literature. Second, they are presented in a broad and rather geographically non-specific manner to be able to give an overview in a single paper. Third, our presentation of these issues is based on basic differences between ethics and law and does not integrate all the intersections and intertwined relations between the two disciplines, since it aims to clarify the distinctions. Fourth, we have chosen not to approach ethical discussions through a single normative approach, which would give importance to a specific classical traditions in ethics (e.g., Aristotle’s virtue ethics or Kantian deontology) or to more contemporary currents such as the ethics of care, but to account for a certain diversity in the presentation of the issues, which can present themselves differently depending on the chosen angle.

## Conclusion

The six issues we highlighted in this article illustrate the intensity and extent to which healthcare professionals are already being affected by the development of AI, and will be even more so in the future. In order for AI to benefit them, as well as patients, healthcare organizations, and society as a whole, we must first know how to identify these issues in practice. It is vital that healthcare professionals can tell whether ethical or legal problems arise while implementing and using AI tools, so they can react to them in the most appropriate way. Such knowledge can guide their usage of AI, allowing them to better adjust to this new technology and to keep a helpful critical lens - notably through a benefit/risk perspective that is already important in the healthcare field. To achieve this, we suggest reviewing the initial and ongoing training of professionals, supporting professionals in their use of AI tools through ethical and regulatory evaluation, and cultivating new reflexes to respond to a “potential risk” in legal or ethical terms.

## Data Availability

We do not analyze or generate any datasets, because our work proceeds within a theoretical approach. One can obtain the relevant materials from the references below.

## Footnotes

1 For instance, Elise A.I. Technologies Corp. specializes in conversational AI solutions. EliseAI offers AI-powered technology that can automate administrative tasks like appointment scheduling and sending payment reminders (SMS, voice, email and web chat formats).

2 For example, Enlitic Inc. is developing deep learning medical tools to streamline radiology diagnoses.

3 For example, Healthee is a company that uses AI to help its team members effectively navigate the coverage and medical treatment options available to them.

4 “Quantity” usually refers to the amount of (massive) data often required to run a system, while “quality” refers to both its accuracy and currency, but also its relevance (the representativeness of the data in relation to the system’s target population, freedom from bias, etc.).

5 We are referring here in part to a liberal conception of privacy as described by Alan Westin or Stanley Benn, who defend the idea of a shield protecting individual autonomy. This is indeed one of the aspects of privacy, which can serve one of the dimensions of individual autonomy in that it creates a space in which individuals feel at ease, whatever the social and political pressures, see: [49]

6 The principle of non-maleficence encompasses privacy (and security) and is, according to the principles of modern medical ethics, a moral standard to be considered. The principle of beneficence encompasses the protection of dignity, from which the protection of privacy also partly derives [54].

7 Some techniques make it difficult to ensure data confidentiality and security [27, 60].

8 Until now, standards-based tools have generally been more prevalent in the sensitive medical sector, where confidentiality of information is essential to the quality of care (e.g. professional and medical secrecy, general obligation of confidentiality of medical records, specific protection laws applicable to the healthcare sector).

9 This may be precisely the objective of the AI system (e.g., to find the risk factors for a disease, or those that lead an individual to buy a particular over-the-counter product), or different data points may be linked before the algorithm even starts running, during the database creation phase.

10 Rumbold and his coauthors show why the coupling of ethnographic, geographic, and genetic data for genomics research is of enormous interest, but can contribute to or directly lead to re-identification [62].

11 Spiekermann and his co-authors present results showing how “engineers” see the need to respect privacy as a barrier to engineering and, by extension, to public utility, and therefore a value which, when integrated into an organization’s ethical standards, is less important than it is a loss of time and autonomy, which sometimes contradict it [63].

12 “Personal,” adjective: “of, relating to, or constituting personal property,” in Merriam-Webster Dictionary.

13 The legislative disposition defines “personal information” as information that identifies, relates to, describes, is reasonably capable of being associated with, or could reasonably be linked, directly or indirectly, with a particular consumer or household.

14 See, for example, the Principle of Purpose limitation in the European Data Protection Regulation (art. 5), the Fair Information Principle, Limiting Use, Disclosure, and Retention in the Canadian Personal Information Protection and Electronic Documents Act (principle 5), as well as the Collection, Use, and Disclosure limitation principle in the HIPAA Privacy Rule.

15 Pesapane et al. (2018) consider that “access to big data of medical images is needed to provide training material to AI devices, so that they can learn to recognise imaging abnormalities. One of the problems is that sensitive data might either be harvested illicitly or collected from unknown sources because of the lack of unique and clear regulations” [68].

16 Few privacy laws refer explicitly to “artificial intelligence,” “machine learning” or other specific AI techniques. However, they do consider AI through the way data is used and the consequences (regulating profiling as analysis and prediction of human behavior; and the subsequent automated decision made without human verification)—see, for example: [67, 69].

17 “Profiling” is now defined and included in the law, but for now the Act only provides for the Attorney General to adopt regulations requiring businesses’ response to access requests to include meaningful information about the logic involved in those decision-making processes, as well as a description of the likely outcome of the process with respect to the consumer [69].

18 A dark sludge can be defined as “an evil nudge […] that can exploit [online consumers’] cognitive biases to persuade them to do something that is undesirable, typically by introducing excessive friction into choice architecture.” Dark sludges include strategies that make consumers’ more opaque, make it harder for them to freely express their preferences, or lead them to take decisions that they would not have taken spontaneously [82].

19 Consent is one of the six legal bases on which the collection of personal data can be legitimate, as stated in Article 6 of the European General Data Protection Regulation.

20 Without establishing consent as an absolute principle, the HIPAA considers that in some situations it is a means of basing the use of health information, and the right to opt out integrated in the California Consumer Privacy Act considers that it must be possible for an individual to refuse the selling or sharing of their information upon request.

21 For example, the European Data Protection Regulation requires information on the existence of automated decision-making or profiling, as well as information useful for understanding the algorithm and its logic and its consequences for the data subject.

22 In France, the “human guarantee principle” was supported by the Ethik-IA group to be integrated into the revision of Article 11 of the French bill relating to bioethics in 2021, taken up in two opinions of the National Ethics Advisory Committee, and involved the exercise of a systematic human review of the real-life conditions of an AI device. This concept was taken up in the proposal for a Regulation of the European Parliament and of the Council in Article 14 (“human control”: COM(2021) 206 final) [91].

23 For instance, Google launched a fairness module in its ML Crash Course in 2018.

24 Efforts to distinguish between prohibited grounds of discrimination are found in numerous international tools such as the Universal Declaration on Human Rights, the International Covenant on Economic, Social and Cultural Rights, the International Covenant on Civil and Political Rights; regional human rights conventions such as the African Charter on Human and People’s Rights, the American Convention on Human Rights and the European Convention on Human Rights; and national legal instruments.

25 Through several case studies (vignettes), the authors demonstrate the tendency to trust the technological tool more when AI is used as a diagnostic assistant, which is mostly the case today [108, 109].

26 For example, the guiding principles value multidisciplinary expertise throughout the product lifecycle so that the benefit/risk balance is assessed not only with regard to validity and clinical efficacy, but also other social risks, confidentiality, representativeness, “human in the loop” performance or at least the role of humans in interpreting the model’s outputs, and user information.

27 For example, advocating the development of “trustworthy AI” would seem to be conceptual nonsense to Dr. Thomas Metzinger, Professor of Philosophy at the University of Mainz in Germany, who argues that machines are not trustworthy as only humans can be trustworthy.
